# Longitudinal assessment of myocardial involvement in PASC-CVS: a single-center study from China based on multiparametric CMR

**DOI:** 10.3389/fcvm.2026.1725291

**Published:** 2026-05-18

**Authors:** Ai Shang, Shan Yang, Jiaye Tao, Jie Shen, Yi Zhan, Yinwen Gan, Zhiyong Zhang, Hang Jin, Fei Shan

**Affiliations:** 1 Department of Radiology, Shanghai Public Health Clinical Center, Fudan University, Shanghai, China; 2 Department of Radiology, Zhongshan Hospital, Fudan University, Shanghai, China

**Keywords:** cardiac magnetic resonance imaging, long coronavirus disease 2019, longitudinal study, multiparametric imaging, post-acute sequelae of SARS-CoV-2 infection with cardiovascular involvement

## Abstract

**Introduction:**

Persistent cardiac symptoms after COVID-19, termed post-acute sequelae of SARS-CoV-2 infection with cardiovascular involvement (PASC-CVS), remain poorly understood. This study aims to reveal myocardial involvement and long-term cardiac magnetic resonance (CMR) changes in PASC-CVS patients, providing insights into disease progression and recovery.

**Methods:**

This prospective single-center study in China enrolled 110 patients with PASC-CVS from March 2023 to April 2024. All participants underwent multiparametric CMR imaging to explore abnormal CMR manifestations in PASC-CVS patients. Clinical symptoms and CMR examinations were collected again one year later. Statistical analysis included the Wilcoxon signed-rank test and logistic regression.

**Results:**

Compared to controls, PASC-CVS patients exhibited significant differences in multiparametric CMR parameters, including Quantification of late gadolinium enhancement (LGE) (%) [odds ratio (OR): 6.36], Global extracellular volume (ECV) (ms) (OR: 1.33), Heart rate (bpm) (OR: 1.08), left ventricular global longitudinal strain (LV GLS) (%) (OR: 1.44), and Global native T1 (ms) (OR: 1.01). The combined multiparametric CMR parameters (Quantification of LGE, Global ECV, Heart rate, LV GLS, Global Post T1, and Global native T1) achieved an area under the curve (AUC) of 0.94, indicating excellent predictive value. At the one-year follow-up (*n* = 101), significant clinical improvement was observed, with a reduction in PASC scores (a 12-point symptom-based threshold framework) from 14.1 to 5.4 (*P* < 0.001). Repeat CMR in 20 patients revealed improvements in right ventricular ejection fraction (RV EF) (52.1% to 59.5%, *P* < 0.001) and global T2 (43.6 ms to 39.5 ms, *P* < 0.001). However, the proportion of patients showing recovery in Quantification of LGE was 0%, suggesting limited clinical significance of its subtle changes.

**Conclusions:**

Our study reveals myocardial involvement in PASC-CVS patients, with follow-up improvements in RV EF and global T2, underscoring the utility of multiparametric CMR in monitoring and managing PASC-CVS.

## 1 Introduction

Since December 2019, the Coronavirus Disease 2019(COVID-19) pandemic has rapidly spread worldwide, with over 777 million cases reported as of January 2025 (1). Studies indicate that 10%–20% of individuals infected with SARS-CoV-2 may develop Post-acute Sequelae of SARS-CoV-2 Infection (PASC), commonly referred to as Long COVID (2, 3). PASC encompasses a range of symptoms that persist or emerge after COVID-19 recovery, typically lasting 4 to 12 weeks or longer (4). The World Health Organization (WHO) defines PASC as the persistence or emergence of new symptoms three months after SARS-CoV-2 infection, lasting at least two months without an alternative explanation (2).

Recent research has advanced the understanding of Long COVID. A prospective cohort study proposed a symptom-based definition, developing a data-driven scoring framework where a PASC score ≥12 classifies patients as PASC-positive (5, 6). Post-Acute Sequelae of SARS-CoV-2 Cardiovascular Syndrome (PASC-CVS) refers to a heterogeneous condition characterized by cardiovascular symptoms, though standard diagnostic methods often lack objective evidence of cardiovascular disease (4).

Although endomyocardial biopsy remains the gold standard for diagnosing myocarditis (6), its limitations include sampling error, limited sensitivity, and inherent risks, including rare mortality (7). Cardiac magnetic resonance (CMR) imaging has emerged as a non-invasive, highly sensitive tool for evaluating myocardial function, structure, and tissue characterization. Beyond conventional measures like ventricular ejection fraction (EF) and volumes, CMR enables myocardial tissue characterization through parametric mapping techniques, which detect diffuse fibrosis, inflammation, and edema (8, 9). Thus, multiparametric CMR may identify patients at high risk of cardiac sequelae and myocardial damage in PASC.

Numerous studies have assessed COVID-19's impact on myocardial tissue using CMR. Puntmann et al. (10) reported cardiac involvement in 78% of 100 recently recovered COVID-19 patients, with ongoing myocardial inflammation in 60% and scarring evident on native T1 and late gadolinium enhancement (LGE) assessment. Similar findings have been reported in smaller studies, including those focusing on recovering athletes. Hanneman et al. (11) found elevated native T1 values associated with cardiac symptoms at 3–6 and 12–18 months post-mild COVID-19.However, these studies were not longitudinal, and the reported frequency of cardiac symptoms was low. In contrast, our study quantifies cardiac symptoms and evaluates their relationship with imaging findings. To date, few long-term longitudinal studies have examined multiparametric CMR findings in PASC-CVS.

Therefore, this study utilizes multiparametric CMR to: (i) explore abnormal imaging characteristics in PASC-CVS patients; and (ii) investigate longitudinal changes in multiparametric CMR findings through a one-year follow-up.

## 2 Materials and methods

This single-center longitudinal cohort study was approved by the institutional research ethics board (Shanghai Public Health Clinical Center). All participants provided written informed consent.

### 2.1 Study population

Participants were prospectively recruited between March 2023 and April 2024. The study included two groups: PASC-CVS patients and controls. Inclusion criteria for the PASC-CVS group were: (i) Age ≥18 years; (ii) Presence of cardiovascular symptoms (e.g., post-exertional malaise, palpitations, chest pain, fatigue) that first occurred within 2 months after SARS-CoV-2 infection. Inclusion criteria for the control group were: (i) No history of SARS-CoV-2 infection; or (ii) Previous SARS-CoV-2 infection but remained asymptomatic. Exclusion criteria for both groups were: (i) Cardiac pacemaker implantation; (ii) Uncontrolled hypertension; (iii) History of severe cardiovascular diseases (e.g., moderate to severe coronary artery disease, myocardial infarction, valvular dysfunction, atrial fibrillation, heart failure, cardiomyopathy); (iv) Severe renal insufficiency (creatinine clearance <30 mL/min/1.73m^2^); (v) Chronic mental illness requiring clinical treatment; (vi) Pregnancy; (vii) Contraindications for MRI; (viii) Severe image artifacts (e.g., significant respiratory motion artifacts).

### 2.2 Data collection and follow-up

Before CMR examination, clinical data were collected, including gender, age, and clinical symptoms (e.g., smell/taste abnormalities, post-exertional malaise, chronic cough, brain fog, thirst, palpitations, chest pain, fatigue, sexual desire/capacity changes, dizziness, gastrointestinal issues, and abnormal movements). For the PASC-CVS group, a follow-up assessment, including clinical data collection and CMR, was conducted 10–14 months after the initial examination using the same protocol.

### 2.3 CMR scanning protocol

Cardiac MRI was performed using two 3.0 T MR scanners (uMR780 and uMR870, United Imaging Healthcare, Shanghai, China). The total scanning duration for each subject is approximately 45 min. A standardized MRI protocol was used to evaluate myocarditis based on alterations in myocardial tissue composition, as previously described (12). Cine imaging was performed using balanced steady-state free precession (bSSFP) sequences to acquire short-axis (SA) and long-axis (LA) images covering the entire heart. Pre- and post-contrast T1 maps were obtained using a Modified Look-Locker inversion recovery (MOLLI) 5(3)3 sequence during diastole, with SA images acquired at basal, mid-ventricular, and apical levels. T2 mapping was performed using T2-prepared bSSFP sequences during diastole at the same locations as T1 mapping. Late gadolinium enhancement (LGE) imaging was conducted after intravenous injection of 0.2 mL/kg gadoterate meglumine (Dotarem, Guerbet, France), with delayed enhancement images acquired 10 min post-injection to assess myocardial delayed enhancement. Multiparametric image analysis was performed to derive global cardiac function metrics, myocardial strain and strain rate, as well as T1, extracellular volume (ECV), and T2 maps. Detailed sequence parameters are provided in Supplementary Table S1.

### 2.4 Cardiac MRI analysis

Image analysis was performed using Circle cvi42 software version 5.13 (Circle, Calgary, AB, Canada). The following parameters were derived: left and right ventricular end-diastolic volume (EDV), end-systolic volume (ESV), stroke volume (SV), cardiac output (CO), ejection fraction (EF), and cardiac index (CI). Myocardial deformation parameters, including global longitudinal, circumferential, and radial peak systolic strains (GLS, GCS, GRS) and peak diastolic strain rates (GLSR, GCSR, GRSR), were analyzed. Tissue characterization parameters, such as T2 values, native T1 values, post-contrast T1 values, and extracellular volume (ECV), were also derived. Endocardial and epicardial contours were manually delineated on T1 and T2 slices, and cvi42 T1 and T2 mapping techniques were used to generate T1 and T2 maps by fitting exponentially recovering (T1) and decaying (T2) curves at the pixel level.

Two radiologists (Y.W.G and A.S., with 1 and 2 years of experience) independently analyzed the MRI studies using Circle cvi42 software, blinded to all clinical information. The presence, location (apical, midventricular, or basal segments), and pattern of late gadolinium enhancement (LGE) were independently assessed by two radiologists (A.S. and F.S., with 2 and 6 years of experience). LGE lesions were quantitatively analyzed using the CVI LGE quantification tool, with lesions defined as areas with signal intensity more than 3 standard deviations above the reference normal myocardium.

### 2.5 Intra- and interobserver reliability

The intra- and interobserver reliability of all CMR parameters was evaluated in 110 PASC-CVS patients and 55 controls. Intraclass correlation coefficients (ICC) were used to assess reliability. Intraobserver reliability was determined by repeated measurements by one radiologist (A.S.) after a minimum 1-month interval, blinded to prior results. Interobserver reliability was assessed independently by another radiologist (Y.W.G.), blinded to the first radiologist's measurements. Results are presented in Supplementary Table S7.

### 2.6 Statistical analysis

Statistical analysis was performed using IBM SPSS Statistics (version 20.0) and RStudio (version 2024.12.0). Categorical data are presented as counts (percentages), and continuous variables as mean ± standard deviation (SD) or median [interquartile range (IQR)]. Normality was assessed using the Shapiro–Wilk test. Proportions were compared using Fisher's exact or *χ*2 tests, as appropriate; for non-paired continuous variables, Student's t-test was used for normally distributed data, while the Mann–Whitney U test was applied for non-normally distributed data. For paired comparisons, McNemar's test was used for categorical variables, and the Wilcoxon signed-rank test was used for continuous variables. Logistic regression was used to analyze CMR parameters, with variables significant at *P* < 0.1 in univariable analysis included in multivariable models. Multivariate logistic regression identified predictors of PASC-CVS. Receiver operating characteristic (ROC) curves were used to evaluate the diagnostic value of single and combined predictors, with area under the curve (AUC) and optimal cutoff points determined by Youden's index. The DeLong test was employed to compare the performance among the parameters of the selected CMR independent predictors and the combined parameter. All tests were two-sided, with *P* < 0.05 considered statistically significant. Intra- and interobserver repeatability were assessed using ICC.

## 3 Results

### 3.1 Baseline clinical characteristics

**Figure 1:**
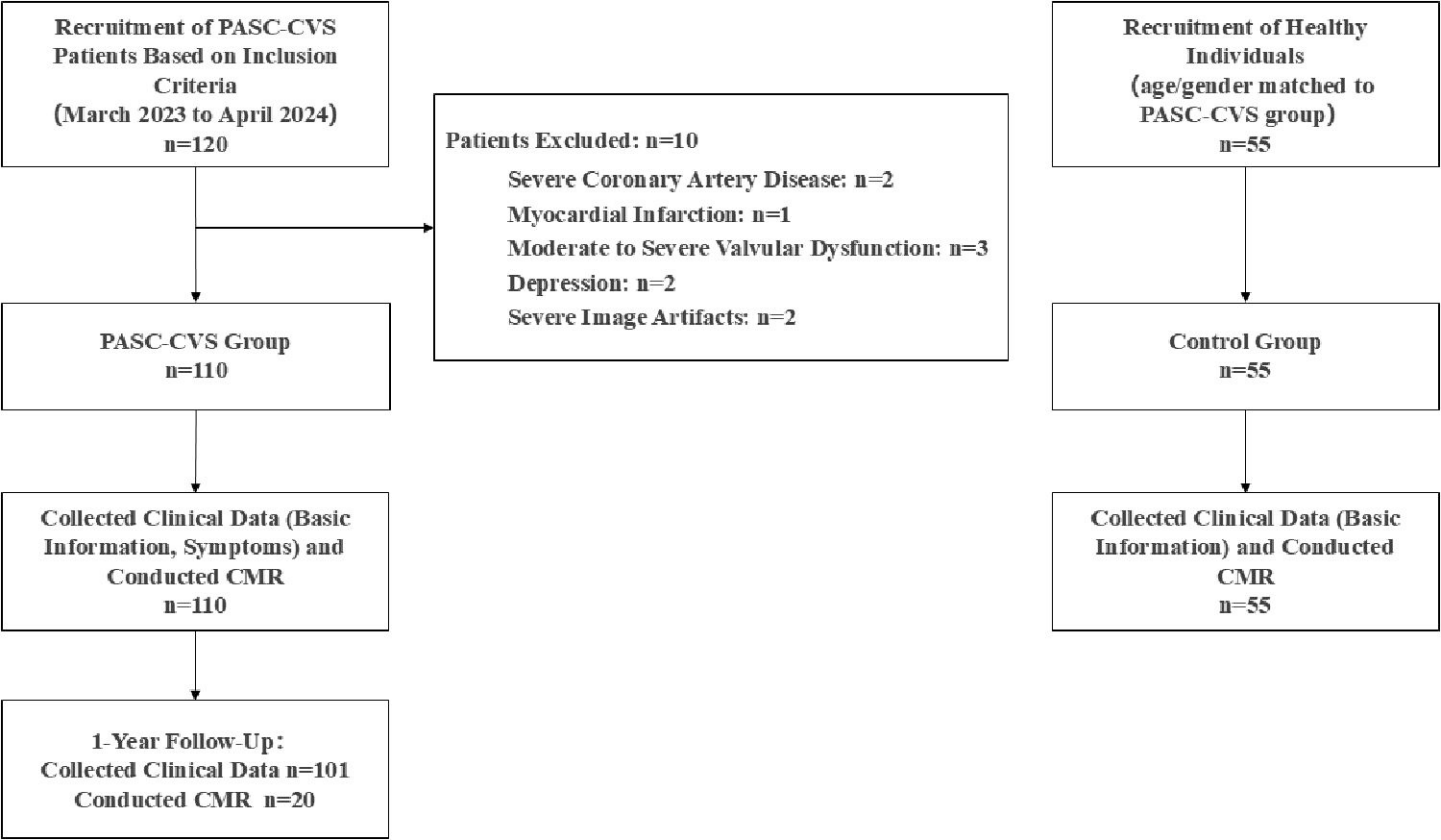
Recruitment pathway for patients in this study. PASC-CVS, Post-Acute Sequelae of SARS-CoV-2 Cardiovascular Syndrome.

Among 120 participants evaluated for eligibility, 10 were excluded (Figure 1). The final cohort included 110 participants, with 35.5% being male (*n* = 39) and a mean age of 42.7 ± 15.6 years. No significant differences in smoking history or other comorbidities were observed between groups. Telephone follow-up was conducted in 101 PASC patients, with 9 patients lost to follow-up due to missing contact information or lack of cooperation. The mean time from virus detection to the initial CMR examination was 204.7 ± 113.0 days (median 169.0 days, IQR 108.3–261.0).

The clinical symptoms of the PASC-CVS subjects, collected during the initial CMR examination, are presented in Supplementary Table S3 and Supplementary Figure S1. The definitions of clinical symptoms are provided in Supplementary Table S2. The total PASC score for the PASC-CVS group was 14.1 ± 4.4 points. The most prevalent symptoms were fatigue (95%), palpitations (94%), and post-exertional malaise (91%).

### 3.2 CMR findings

Among the 55 controls, 15 had no history of SARS-CoV-2 infection and 40 had prior infection but remained asymptomatic. Subgroup analysis showed no significant differences in CMR parameters between these two control subgroups (Supplementary Table S4). The global MRI parameters are summarized in Tables 1 and S5. Compared to controls, the PASC-CVS group exhibited significant differences in key cardiac parameters. Specifically, the PASC-CVS group had elevated heart rate (74.4 ± 12.7 bpm vs. 66.4 ± 9.4 bpm, *P* < 0.001), and Global ECV values (31.6 ± 2.6% vs. 28.9 ± 2.2%, *P* < 0.001). Additionally, LV GCS (−19.7 ± 3.1% vs. −20.9 ± 2.3%, *P* = 0.008) and LV GLS (−15.1 ± 2.5% vs. −16.2 ± 1.9%, *P* = 0.010) were impaired in the PASC-CVS group. Quantification of LGE was significantly higher in the PASC-CVS group (0.7 ± 0.3% vs. 0.3 ± 0.2%, *P* < 0.001), and the prevalence of LGE was also significantly increased (71.8% vs. 37.0%, *P* < 0.001).

**Table 1:** Baseline characteristics and CMR findings of PASC-CVS compared to control.

Characteristic	PASC-CVS(*n* = 110)	Control(*n* = 55)	*P* value
Age（years）	42.65 ± 15.55	42.87 ± 13.89	0.740
Male Gender（N，%）	39 (35.45)	25 (45.45)	0.283
BMI（kg/m2）	23.02 ± 3.61	23.52 ± 3.61	0.397
Smoking history（N，%）	19 (17.27)	9 (16.36)	1.000
Comorbidities			
Hypertension（N，%）	17 (15.45)	8 (14.55)	1.000
Diabetes（N，%）	7 (6.36)	2 (3.64)	0.719
Hyperlipidemia（N，%）	14 (12.73)	4 (7.27)	0.428
Heart Failure（N，%）	0 (0.00)	0 (0.00)	NA
Severe coronary artery disease（N，%）	0 (0.00)	0 (0.00)	NA
Other Heart Diseases（N，%）	0 (0.00)	0 (0.00)	NA
History of Coronary Artery Surgery（N，%）	0 (0.00)	0 (0.00)	NA
History of Psychotropic Medication（N，%）	2 (1.82)	0 (0.00)	0.553
Depression（N，%）	0 (0.00)	0 (0.00)	NA
Anxiety（N，%）	13 (11.82)	0 (0.00)	0.005*
Blood biomarkers			
Troponin-positive（N，%）	1（0.91）	NA	NA
COVID-19 vaccination			
At least three dose prior to MRI（N，%）	99 (90.00)	54 (98.18)	0.063
Cardiac MRI			
Days from virus detection to CMR (days)	204.73 ± 112.98	NA	NA
Days from virus detection to CMR (days)^#^	169.00 (108.25–261.00)	NA	NA
uMR 780（N，%）	84 (76.36)	36 (65.45)	0.194
LV EDV（mL）	118.38 ± 27.17	121.17 ± 23.74	0.454
LV ESV（mL）	50.45 ± 20.71	48.54 ± 12.12	0.726
LV SV（mL）	67.92 ± 15.03	72.63 ± 14.97	0.139
LV CO（/min）	5.02 ± 1.35	4.79 ± 1.05	0.236
LV EF（%）	57.83 ± 7.38	60.05 ± 5.50	0.150
LV CI（/min/m2）	2.91 ± 0.68	2.77 ± 0.48	0.197
RV EDV（mL）	114.53 ± 27.95	125.15 ± 30.45	0.037*
RV ESV（mL）	56.06 ± 20.86	57.42 ± 17.27	0.414
RV SV（mL）	58.47 ± 15.98	67.73 ± 16.99	0.001*
RV CO（/min）	4.33 ± 1.38	4.47 ± 1.22	0.435
RV EF（%）	51.51 ± 9.86	54.38 ± 6.30	0.091
RV CI（/min/m2）	2.51 ± 0.75	2.57 ± 0.54	0.504
Heart rate (bpm)	74.40 ± 12.71	66.41 ± 9.37	<0.001*
Global native T1（ms）	1,153.53 ± 66.53	1,132.86 ± 49.10	0.076
Global post T1（ms）	454.75 ± 58.60	384.98 ± 73.74	<0.001*
Global ECV（%）	31.56 ± 2.59	28.86 ± 2.22	<0.001*
Global T2（ms）	42.24 ± 3.56	42.45 ± 2.74	0.778
LV GRS（%）	35.42 ± 9.04	35.91 ± 7.63	0.730
LV GCS（%）	−19.65 ± 3.09	−20.87 ± 2.25	0.008*
LV GLS（%）	−15.12 ± 2.49	−16.23 ± 1.89	0.010*
LV sGRSR (/s)	2.29 ± 0.81	2.35 ± 1.07	0.403
LV sGCSR (/s)	−1.08 ± 0.23	−1.06 ± 0.18	0.553
LV sGLSR (/s)	−0.90 ± 0.22	−0.90 ± 0.27	0.550
LGE（N，%）	79 (71.82)	17 (36.96)	<0.001*
Quantification of LGE (%)	0.73 ± 0.26	0.34 ± 0.16	<0.001*
Distribution of LGE（N，%）			
Basal	72 (65.45)	5 (10.87)	<0.001*
Mid	33 (30.00)	13 (28.26)	0.980
Apical	5 (4.55)	1 (2.17)	0.671
subepicardial	0 (0.00)	0 (0.00)	NA
epicardial	4 (3.64)	0 (0.00)	0.320
mid-myocardial layer	79 (71.82)	17 (36.96)	<0.001*

Values are presented as mean ± standard deviation or number (%) unless otherwise specified; ^#^, Data are expressed as the median, with the interquartile range in parentheses, for continuous variables; *, P＜0.05 statistically significant; BMI, body mass index [weight [kg]/height^2^ [m^2^]]; LV, left ventricular; EDV, end-diastolic volume; ESV, end-systolic volume; SV, stroke volume; CO, cardiac output; EF, ejection fraction; CI, cardiac index; RV, right ventricular; Global native T1, global native T1 relaxation time; Global post T1, global post-contrast T1 relaxation time; Global ECV, global extracellular volume fraction; Global T2, global T2 relaxation time; GRS, global radial strain; GCS, global circumferential strain; GLS, global longitudinal strain; sGRSR, systolic radial strain rate; sGCSR, systolic circumferential strain rate; sGLSR, systolic longitudinal strain rate; LGE, late gadolinium enhancement.

**Figure 2:**
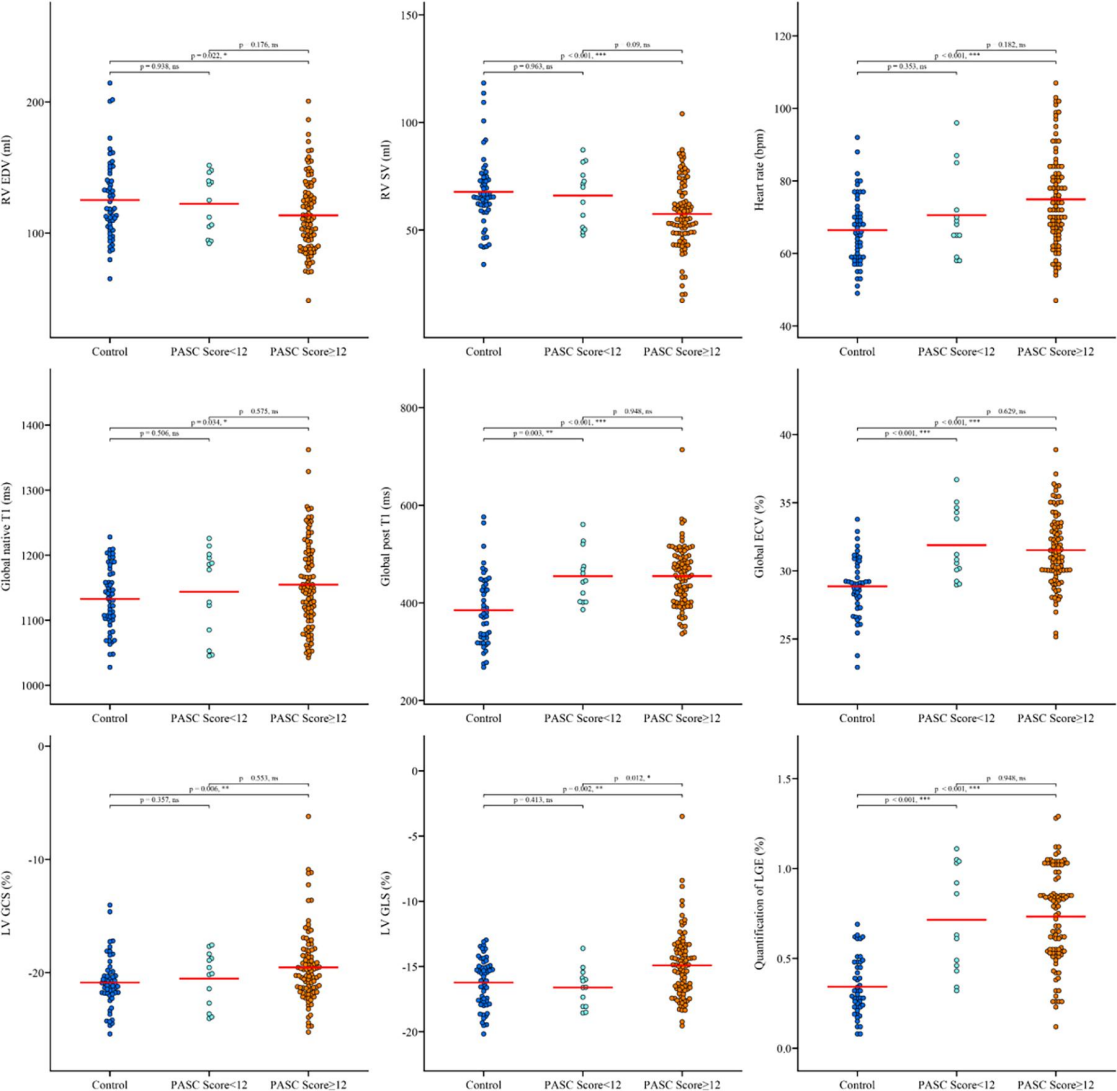
Comparison of CMR parameters among control, PASC score <12, and PASC score ≥12 groups. Distribution of CMR parameters among three groups: Control (*n* = 55), PASC Score <12 (*n* = 13), and PASC Score ≥12 (*n* = 97). Parameters with statistical differences between Control and PASC Score ≥ 12 are shown in this figure, with *P* values <0.05 indicating statistical significance. Significant differences are marked with asterisks, with the number of asterisks corresponding to the level of significance (e.g., *p < 0.05, **p < 0.01, ***p < 0.001). The remaining parameters without statistical significance are displayed in Supplementary Figure S2. Abbreviations are consistent with those used in Table 1.

Furthermore, some CMR parameters showed significant differences between controls and the PASC Score ≥ 12 group but not between controls and the PASC Score < 12 group. For example, heart rate was significantly higher in the PASC Score ≥ 12 group compared to controls (74.9 ± 12.8 bpm vs. 66.4 ± 9.4 bpm, *P* < 0.001), but not in the PASC Score < 12 group (70.5 ± 11.8 bpm vs. 66.4 ± 9.4 bpm, *P* = 0.180). Similarly, Global native T1 values were elevated in the PASC Score ≥ 12 group (1,154.8 ± 66.7 ms vs. 1,132.9 ± 49.1 ms, *P* = 0.034) but not in the PASC Score < 12 group (1,143.8 ± 67.2 ms vs. 1,132.9 ± 49.1 ms, *P* = 0.506). LV GCS (−19.5 ± 3.2% vs. −20.9 ± 2.3%, *P* = 0.006) and LV GLS (−14.9 ± 2.5% vs. −16.2 ± 1.9%, *P* = 0.002) were also significantly impaired in the PASC Score ≥ 12 group compared to controls, but not in the PASC Score < 12 group (LV GCS: −20.5 ± 2.4% vs. −20.9 ± 2.3%, *P* = 0.357; LV GLS: −16.6 ± 1.5% vs. −16.2 ± 1.9%, *P* = 0.505) (Figures 2, S2).

### 3.3 Logistic regression and ROC curve analysis

To assess the ability of CMR parameters to distinguish PASC-CVS from controls, logistic regression and receiver operating characteristic (ROC) curve analyses were performed. Multivariate analysis identified six independent predictors of PASC-CVS: Quantification of LGE (OR: 6.36, 95% CI: 3.05–15.86, *P* < 0.001), Global ECV (OR: 1.33, 95% CI: 1.04–1.78, *P* = 0.034), Global post T1 (OR: 1.02, 95% CI: 1.01–1.03, *P* = 0.002), Global native T1 (OR: 1.01, 95% CI: 1.00–1.02, *P* = 0.049), LV GLS (OR: 1.44, 95% CI: 1.09–1.97, *P* = 0.015), and Heart rate (OR: 1.08, 95% CI: 1.02–1.15, *P* = 0.014) (Table 2).

**Table 2:** Univariate and multivariate logistic regression analysis of multiparametric CMR in PASC-CVS.

Variable	Univariate			Multivariate		
	OR	95% CI	*P* value	OR	95% CI	*P* value
LV EDV（mL）	1.00	0.98–1.01	0.516			
LV ESV（mL）	1.01	0.99–1.03	0.533			
LV SV（mL）	0.98	0.96–1.00	0.062			
LV CO（l/min）	1.17	0.9–1.55	0.266			
LV EF（%）	0.95	0.89–1.00	0.053			
LV CI（l/min/m2）	1.51	0.87–2.71	0.154			
RV EDV（mL）	0.99	0.98–1.00	0.030			
RV ESV（mL）	1.00	0.98–1.01	0.674			
RV SV（mL）	0.97	0.94–0.99	0.001*	0.97	0.93–1	0.083
RV CO（l/min）	0.92	0.72–1.18	0.511			
RV EF（%）	0.96	0.92–1.00	0.055			
RV CI（l/min/m2）	0.88	0.54–1.44	0.614			
Heart rate (bpm)	1.07	1.03–1.11	<0.001*	1.08	1.02–1.15	0.014*
Global native T1（ms）	1.01	1.00–1.01	0.045	1.01	1.00–1.02	0.049*
Global post T1（ms）	1.02	1.01–1.02	<0.001*	1.02	1.01–1.03	0.002*
Global ECV（%）	1.55	1.31–1.87	<0.001*	1.33	1.04–1.78	0.034*
Global T2（ms）	0.98	0.89–1.08	0.704			
LV GRS（%）	0.99	0.96–1.03	0.728			
LV GCS（%）	1.19	1.05–1.38	0.012*			
LV GLS（%）	1.27	1.08–1.51	0.005*	1.44	1.09–1.97	0.015*
LV sGRSR (/s)	0.93	0.65–1.33	0.675			
LV sGCSR (/s)	0.67	0.13–3.01	0.611			
LV sGLSR (/s)	1.07	0.26–4.16	0.924			
Quantification of LGE (%)	5.94	3.49–11.14	<0.001*	6.36	3.05–15.86	<0.001*

*, P＜0.05 Statistically significant; OR, Odds Ratio; CI, Confidence Interval; Abbreviations are consistent with those used in Table 1.

**Figure 3:**
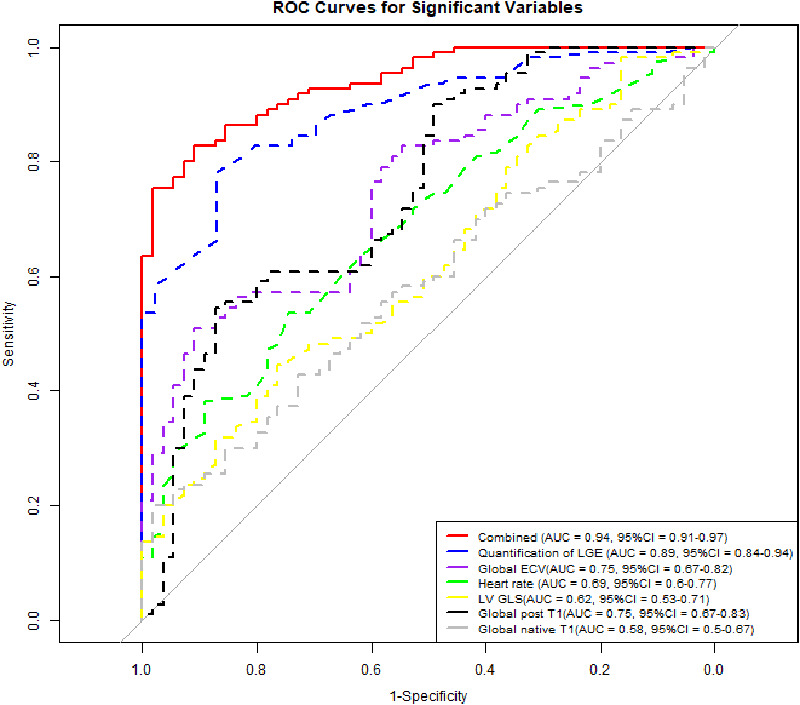
ROC curves of six CMR parameters and combined model this ROC curve demonstrates the ability of six CMR parameters, which were identified as independent predictors through multivariate analysis, and their combined model to distinguish PASC-CVS from the control group: combined (red solid), quantification of LGE (blue dashed), global ECV (purple dashed), heart rate (green dashed), LV GLS (yellow dashed), global post T1 (black dashed), and global native T1 (gray dashed). The red solid curve, representing the combined model, shows the highest AUC of 0.94, indicating excellent discrimination. The blue dashed curve, for Quantification of LGE, has an AUC of 0.89. The purple, green, yellow, black, and gray dashed curves represent Global ECV, Heart rate, LV GLS, Global Post T1, and Global native T1 with AUCs of 0.75, 0.69, 0.62, 0.75, and 0.58, respectively.

Compared with the ROC curves of other independent predictors, the Combined parameter demonstrated superior performance in distinguishing PASC-CVS from controls (*P* < 0.05) (Supplementary Table S6), with an AUC of 0.94 (95% CI: 0.91–0.97, *P* < 0.001), sensitivity of 83.0%, and specificity of 91.0%. Among individual parameters, Quantification of LGE exhibited an AUC of 0.89 (95% CI: 0.84–0.94, *P* < 0.001), sensitivity of 78.0%, specificity of 87.0%, and a cutoff value of 0.52%. Global ECV showed an AUC of 0.75 (95% CI: 0.67–0.82, *P* < 0.001), sensitivity of 51.0%, specificity of 91.0%, and a cutoff value of 31.15%. Global post T1 had an AUC of 0.75 (95% CI: 0.67–0.83, *P* < 0.001), sensitivity of 55.0%, specificity of 87.0%, and a cutoff value of 450.85 ms. Global native T1 showed an AUC of 0.58 (95% CI: 0.50–0.67, *P* = 0.045), sensitivity of 20.0%, specificity of 98.0%, and a cutoff value of 1,210.39 ms. LV GLS exhibited an AUC of 0.62 (95% CI: 0.53–0.71, *P* = 0.005), sensitivity of 45.0%, specificity of 76.0%, and a cutoff value of −14.91%. Heart rate demonstrated an AUC of 0.69 (95% CI: 0.60–0.77, *P* < 0.001), sensitivity of 54.0%, specificity of 75.0%, and a cutoff value of 71.50 bpm (Figure 3, Table 3).

**Table 3:** Receiver operating characteristic curve analysis of CMR independent predictors and combined parameters in distinguishing PASC-CVS from control.

Parameter	AUC	95% CI	Sensitivity	Specificity	Cutoff value
Combined	0.94	0.91–0.97	0.83	0.91	0.70
Quantification of LGE (%)	0.89	0.84–0.94	0.78	0.87	0.52
Global ECV（ms）	0.75	0.67–0.82	0.51	0.91	31.15
Global post T1（ms）	0.75	0.67–0.83	0.55	0.87	450.85
Heart rate (bpm)	0.69	0.60–0.77	0.54	0.75	71.50
LV GLS (%)	0.62	0.53–0.71	0.45	0.76	−14.91
Global native T1（ms）	0.58	0.5–0.67	0.2	0.98	1,210.39

ROC, receiver operating characteristic; AUC, area under the curve; GLS, global longitudinal strain; ECV, extracellular volume; LGE, late gadolinium enhancement; LV, left ventricular.

### 3.4 Clinical and CMR findings one year later

**Figure 4:**
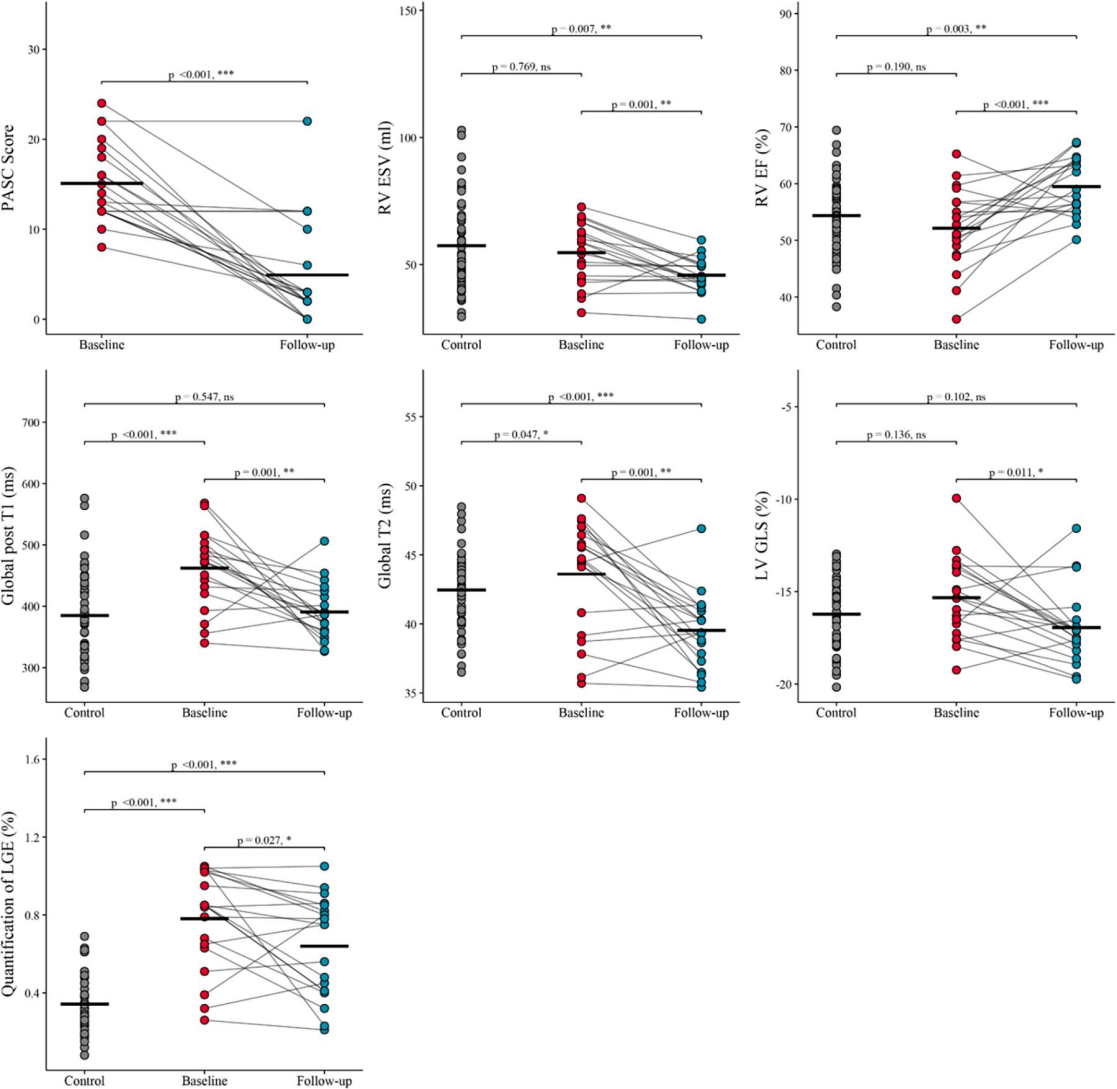
Comparison of CMR parameters among the control, baseline, and follow-up. Distribution of CMR parameters among three groups: Control (*n* = 55), Baseline (*n* = 20), and Follow-up (*n* = 20). Parameters with statistical differences between Baseline and Follow-up are shown in this figure, with *P* values <0.05 indicating statistical significance. Significant differences are marked with asterisks, with the number of asterisks corresponding to the level of significance (e.g., *p < 0.05, **p < 0.01, ***p < 0.001). The remaining parameters without statistical significance are displayed in Supplementary Figure S3. Abbreviations are consistent with those used in Table 1.

**Figure 5:**
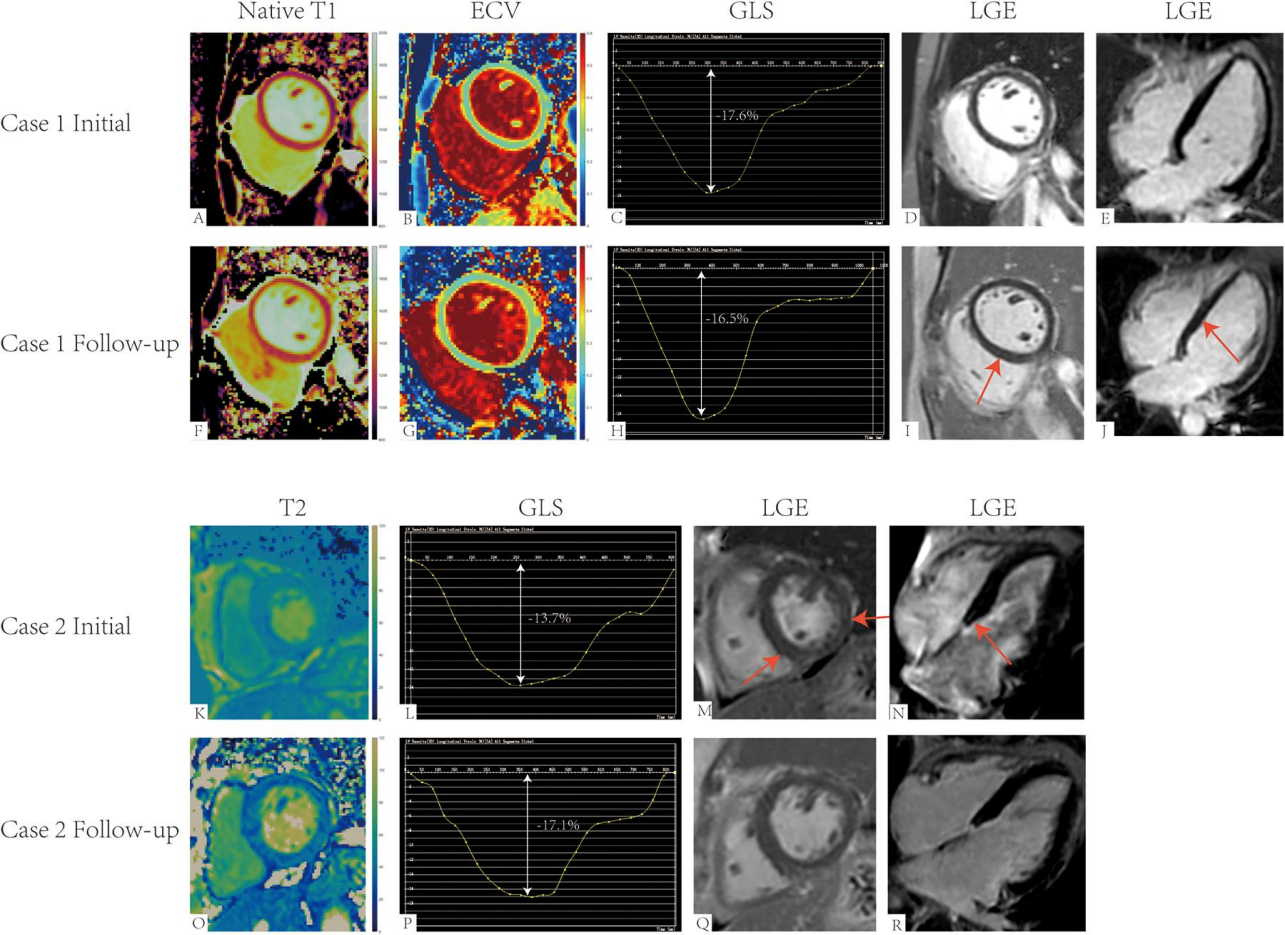
Cardiac magnetic resonance (CMR) imaging of post-acute sequelae of SARS-CoV-2 infection with cardiovascular symptoms (PASC-CVS) patients: initial and follow-up. **(A–J)** CMR images of a 31-year-old female PASC-CVS patient with a stable PASC score of 12 at both initial and follow-up. Initial CMR showed native T1 mapping values of 1,202 ms, extracellular volume (ECV) mapping values of 32%, and global longitudinal strain (GLS) of −17.6%. Follow-up CMR revealed an increase in native T1 to 1,244 ms, ECV to 33%, and a slight reduction in GLS to −16.5%. New mid-myocardial late gadolinium enhancement (LGE) was observed in the interventricular septum on follow-up imaging. Images from two different projections (short-axis and four-chamber) are shown to better illustrate the LGE lesion **(E,J)**. **(K–R)** CMR images of a 70-year-old female PASC-CVS patient with significant clinical improvement, as evidenced by a reduction in PASC score from 16 at initial to 2 at follow-up. Initial CMR showed T2 mapping values of 47 ms, non-ischemic mid-myocardial LGE in the interventricular septum and lateral wall, and GLS of −13.7%. Follow-up CMR demonstrated a reduction in T2 mapping values to 37 ms, complete resolution of the previously observed LGE, and improvement in GLS to −17.1%. The LGE lesion is depicted in both short-axis and four-chamber views **(*N*,R)**.

One year later, with a median follow-up time of 371.00 days (340.00–409.00), significant improvements were observed in the PASC score among patients (*n* = 101), decreasing from 14.1 ± 4.4 to 5.4 ± 5.1, although 9.9% of patients showed no improvement (Supplementary Table S3). Among the 20 patients who underwent repeat CMR, several cardiac parameters demonstrated significant changes (Table 4, Figures 4, S3). Notably, RV EF increased from 52.1 ± 6.8% to 59.5 ± 5.0% (*p* < 0.001), and Global T2 values decreased from 43.6 ± 4.0 ms to 39.5 ± 2.6 ms (*p* < 0.001), while LV GLS improved from −15.3 ± 2.1% to −17.0 ± 2.0% (*p* = 0.011). The Quantification of LGE decreased from 0.78 ± 0.24% to 0.64 ± 0.25% (*p* = 0.027), with Cohen's d values for Quantification of LGE and LV GLS being less than 0.8, indicating moderate effect sizes. The proportions of patients showing recovery (defined as values below the mean of the control group) for RV EF, Global T2, LV GLS, and Quantification of LGE were 85%, 90%, 80%, and 0%, respectively. Among patients who underwent follow-up CMR, those without clinical improvement exhibited worsening CMR findings, while patients with clinical improvement showed corresponding improvements in CMR parameters (Figure 5).

**Table 4:** Characteristics and CMR findings of PASC-CVS patients at one-year follow-up.

Characteristic	Baseline(*n* = 20)	Follow-up(*n* = 20)	*P* value (Cohen's d)
Median Follow-Up Time (days)^#^	NA	416.00 (402.00–429.00)	NA
PASC Score	15.10 ± 4.29	4.90 ± 5.62	<0.001* (1.52)
Improvement（N，%）	NA	17 (85.00)	NA
LV EDV（mL）	120.36 ± 21.80	113.77 ± 15.77	0.294 (0.34)
LV ESV（mL）	49.25 ± 9.87	45.81 ± 6.62	0.076 (0.37)
LV SV（mL）	71.12 ± 14.01	67.96 ± 12.78	0.522 (0.23)
LV CO（l/min）	5.08 ± 0.84	4.82 ± 0.88	0.294 (0.24)
LV EF（%）	59.00 ± 4.08	59.48 ± 4.97	0.546 (−0.09)
LV CI（l/min/m2）	2.83 ± 0.46	2.75 ± 0.49	0.571 (0.12)
RV EDV（mL）	115.27 ± 24.46	113.77 ± 15.77	0.841 (0.06)
RV ESV（mL）	54.71 ± 11.60	45.81 ± 6.62	0.001* (0.84)
RV SV（mL）	60.56 ± 16.60	67.96 ± 12.78	0.097 (−0.36)
RV CO（l/min）	4.34 ± 1.14	4.82 ± 0.88	0.165 (−0.32)
RV EF（%）	52.12 ± 6.83	59.48 ± 4.97	<0.001* (−0.90)
RV CI（l/min/m2）	2.42 ± 0.62	2.75 ± 0.49	0.143 (−0.37)
Heart rate (bpm)	72.75 ± 11.90	71.70 ± 10.62	0.869 (0.07)
Global native T1（ms）	1,125.18 ± 42.86	1,129.49 ± 41.91	0.522 (−0.12)
Global post T1（ms）	461.91 ± 61.02	390.52 ± 44.06	0.001* (0.86)
Global ECV（%）	31.06 ± 1.52	30.05 ± 1.47	0.090 (0.51)
Global T2（ms）	43.61 ± 3.95	39.52 ± 2.60	0.001* (0.97)
LV GRS（%）	33.34 ± 5.70	37.12 ± 7.88	0.053 (−0.45)
LV GCS（%）	−20.26 ± 2.38	−20.44 ± 1.89	0.784 (0.10)
LV GLS（%）	−15.33 ± 2.14	−16.95 ± 1.96	0.011* (0.60)
LV sGRSR (/s)	2.25 ± 0.69	3.03 ± 1.67	0.097 (−0.41)
LV sGCSR (/s)	−1.09 ± 0.15	−1.06 ± 0.21	0.571 (−0.15)
LV sGLSR (/s)	−0.93 ± 0.18	−1.00 ± 0.16	0.216 (0.32)
LGE（N，%）	17 (85.0)	16 (80.0)	0.707
Quantification of LGE (%)	0.78 ± 0.24	0.64 ± 0.25	0.027 * (0.54)
Distribution of LGE (N, %)			
Basal	16 (80.0)	15 (75.0)	0.739
Mid	7 (35.0)	6 (30.0)	0.108
Apical	1 (5.0)	0 (0.0)	0.317
subepicardial	0 (0.0)	0 (0.0)	NA
epicardial	0 (0.0)	0 (0.0)	NA
mid-myocardial layer	17 (85.0)	16 (80.0)	0.317

Values are presented as mean ± standard deviation or number (%) unless otherwise specified; ^#^, presented as medians (IQR); *, P＜0.05 Statistically significant; Abbreviations are consistent with those used in Table 1.

## 4 Discussion

This study is one of the few longitudinal investigations aimed at revealing subtle CMR abnormalities in PASC-CVS and conducting follow-up assessments. Multivariate analysis identified six independent predictors of PASC-CVS: Quantification of LGE, Global ECV, Global post T1, Global native T1, LV GLS, and Heart rate. The combination of these predictors demonstrated significant predictive value, highlighting the potential utility of multiparametric CMR as a valuable source of imaging biomarkers for PASC-CVS. At the one-year follow-up, overall improvement in clinical symptoms and a reduction in CMR abnormalities were observed, notably in RV EF, Global T2, and LV GLS, with 85%, 90%, and 80% of PASC-CVS patients showing improvement in these parameters, respectively. This underscores the value of multiparametric CMR in guiding rehabilitation strategies and optimizing long-term patient management.

This study employed a broad definition of PASC based on the presence of cardiovascular symptoms after SARS-CoV-2 infection. The 12-point PASC score and its ≥12 threshold were applied *post hoc* for exploratory stratification of symptom burden. We acknowledge that this scoring system was derived from non-Chinese populations, and its external validity in Chinese cohorts should be interpreted cautiously. For instance, the researchers who developed the PASC scoring system reported 41% of patients in the original study experienced loss of or change in smell or taste, whereas only 4.8% of patients reported such symptoms in a study conducted in China (13). Future studies validating symptom-based PASC definitions in diverse ethnic and geographic populations are warranted to enhance cross-cohort comparability.

LV GLS was significantly impaired in the PASC-CVS group compared to the control group (−15.1% vs. −16.2%, *P* = 0.010), while no significant difference was observed in LV EF. This suggests that LV GLS may be a more sensitive indicator of subclinical cardiac dysfunction in PASC-CVS patients (14, 15). The increased heart rate (74.4 bpm vs. 66.4 bpm, *P* < 0.001) may be associated with clinical symptoms such as palpitations. Patients in the PASC-CVS group exhibited myocardial fibrosis, as evidenced by elevated Global ECV values (31.6% vs. 28.9%, *P* < 0.001). These findings are consistent with previous studies (4, 10, 15–18). Importantly, these objective CMR abnormalities provide a potential pathophysiological basis for the highly prevalent symptoms of post-exertional malaise (91%) and fatigue (95%) observed in our cohort, highlighting the clinical significance of subclinical LV GLS impairment and elevated native T1 in PASC-CVS. In our study, the majority of patients displayed LGE in a non-ischemic distribution pattern, was predominantly localized at the basal and mid-ventricular segments of the LV, with a subepicardial and/or mid-wall distribution (19, 20). The prevalence of LGE was 70.9%, consistent with the 78.8% reported by Popovic et al. (21).

Furthermore, some CMR parameters showed significant differences between controls and the PASC Score ≥ 12 group but not between controls and the PASC Score < 12 group. This may suggest that the severity of clinical symptoms in PASC-CVS is associated with the extent of CMR abnormalities. We acknowledge that the clinical severity of SARS-CoV-2 infection has varied over time due to viral mutations, which may influence the generalizability of our findings to future variants.

Our study underscores the roles of Quantification of LGE, Global ECV, Global post T1, Global native T1, LV GLS, and heart rate as independent predictive factors, which could aid in the more precise identification of high-risk patients in clinical practice. The ROC analysis revealed the Combined parameter as the most discriminative, with the highest AUC of 0.94. Quantification of LGE followed with an AUC of 0.89, while Global ECV and Global post T1 showed AUCs of 0.75. LGE is indicative of tissue inflammation, necrosis, and fibrosis, suggesting that chronic inflammation is a primary mechanism of direct myocardial injury caused by COVID-19 (22–24). Additionally, earlier studies on other types of viral myocarditis have demonstrated that LGE carries prognostic significance, often correlating with a worse prognosis (25–27).

Our findings are consistent with previous studies (11, 28) and demonstrate an overall improvement in clinical symptoms among PASC patients, with a reduction in PASC scores from 14.1 ± 4.4 at baseline to 5.4 ± 5.1 at follow-up (*n* = 101). However, 9.9% of patients still showed no improvement, highlighting the necessity for repeat CMR assessments to evaluate the progression of myocardial involvement. However, the rate of follow-up CMR completion was relatively low, with only 20 out of 101 participants undergoing a second CMR examination. This high dropout rate was primarily due to patients’ reluctance to repeat CMR after symptom improvement, as well as the geographical challenges posed by the inclusion of patients from across the country, many of whom were unwilling to return to our center for follow-up.

Significant differences in cardiac function, including RV EF and LV GLS, were observed on repeat CMR. These findings are consistent with the study by Puntmann et al., who reported follow-up CMR findings at a median of 329 days after the initial CMR and observed a notable increase in RV EF (from 54.0 ± 5.6% to 55.4 ± 5.6%) (16). Additionally, improvements in T2 values were observed, indicating a reduction in myocardial edema. This finding aligns with the understanding that in PASC patients, myocardial edema tends to resolve over time as the acute phase subsides (21, 29). However, our study demonstrated a significant reduction in quantitative LGE at the one-year follow-up, which contrasts with previous reports suggesting that LGE without accompanying edema on a 6-month follow-up CMR indicates definitive fibrosis and irreversible myocardial injury (22, 30, 31). Nevertheless, this discrepancy may be attributed to the effect size of Quantification of LGE being less than 0.8, and the proportion of patients showing recovery was 0%, suggesting that the subtle changes in Quantification of LGE during follow-up may lack clinical significance. The subtle quantitative reduction likely reflects technical variability of the >3SD threshold or mild interstitial signal changes, rather than biological recovery of established fibrosis.

Overall, these longitudinal findings provide a reassuring prognostic outlook. The concurrent improvement in both symptoms and objective CMR parameters, together with patients’ reluctance to undergo repeat CMR due to symptom resolution, suggests that PASC-CVS follows a benign, self-limiting course in most patients without severe baseline disease.

## 5 Limitations

Our study has several limitations. First, it is a single-center study with a modest sample size, necessitating larger multicenter studies to validate and generalize our findings. Second, the reported prevalence of symptoms may be influenced by self-selection bias due to recruitment through invitations, potentially affecting cohort representativeness. Third, the follow-up CMR subgroup was limited in size and potentially subject to selection bias, which may have influenced the observed recovery patterns. Fourth, the absence of a histopathologic reference standard, such as endomyocardial biopsy, limits direct correlation of our pathophysiologic conclusions with histopathologic results. Future studies incorporating such standards are needed to further elucidate the underlying mechanisms.

## 6 Conclusion

Our study reveals myocardial involvement in PASC-CVS and supports the potential utility of multiparametric CMR, particularly through predictors such as Quantification of LGE, Global ECV, Global post T1, Global native T1, LV GLS, and heart rate, as a valuable source of imaging biomarkers for PASC-CVS. The observed improvements in clinical symptoms and CMR abnormalities (notably RV EF, Global T2, and LV GLS) at the one-year follow-up highlight the role of CMR in guiding long-term patient management. However, the persistence of symptoms in a subset of patients and the challenges in follow-up CMR completion underscore the need for further longitudinal studies to better understand the progression and prognostic implications of these findings. Future research should focus on validating these results in larger, multicenter cohorts and integrating histopathologic correlations to further elucidate the underlying mechanisms of myocardial injury in PASC-CVS.

## Data Availability

The original contributions presented in the study are included in the article/Supplementary Material, further inquiries can be directed to the corresponding author/s.

## References

[1] World Health Organization. COVID-19 cases dashboard (2025). Available online at: https://data.who.int/dashboards/covid19/cases?n=c [Accessed January 10, 2025].

[2] World Health Organization. Post COVID-19 condition (Long COVID) (2022). Available online at: https://www.who.int/europe/news-room/fact-sheets/item/post-covid-19-condition [Accessed January 10, 2025].

[3] Zhang D Chen C Xie Y Zhou S Li D Zeng F Prevalence and risk factors of long COVID-19 persisting for 2 years in hainan province: a population-based prospective study. Sci Rep. (2025) 15:369. 10.1038/s41598-024-84598-4 39747631 PMC11696313

[4] Gluckman TJ Bhave NM Allen LA Chung EH Spatz ES Ammirati E 2022 ACC expert consensus decision pathway on cardiovascular sequelae of COVID-19 in adults: myocarditis and other myocardial involvement, post-acute sequelae of SARS-CoV-2 infection, and return to play. J Am Coll Cardiol. (2022) 79:1717–56. 10.1016/j.jacc.2022.02.003 35307156 PMC8926109

[5] Thaweethai T Jolley SE Karlson EW Levitan EB Levy B McComsey GA Development of a definition of post-acute sequelae of SARS-CoV-2 infection. JAMA. (2023) 329:1934–46. 10.1001/jama.2023.8823 37278994 PMC10214179

[6] Aretz HT. Myocarditis: the Dallas criteria. Hum Pathol. (1987) 18:619–24. 10.1016/S0046-8177(87)80363-5 3297992

[7] Vidusa L Kalejs O Maca-Kaleja A Strumfa I. Role of endomyocardial biopsy in diagnostics of myocarditis. Diagnostics (Basel). (2022) 12:2104. 10.3390/diagnostics12092104 36140505 PMC9497694

[8] aus dem Siepen F Buss SJ Messroghli D Andre F Lossnitzer D Seitz S T1 mapping in dilated cardiomyopathy with cardiac magnetic resonance: quantification of diffuse myocardial fibrosis and comparison with endomyocardial biopsy. Eur Heart J Cardiovasc Imaging. (2015) 16:210–16. 10.1093/ehjci/jeu183 25246502

[9] Galea N Marchitelli L Pambianchi G Catapano F Cundari G Birtolo LI T2-mapping increase is the prevalent imaging biomarker of myocardial involvement in active COVID-19: a cardiovascular magnetic resonance study. J Cardiovasc Magn Reson. (2021) 23:68. 10.1186/s12968-021-00764-x 34107985 PMC8189727

[10] Puntmann VO Carerj ML Wieters I Fahim M Arendt C Hoffmann J Outcomes of cardiovascular magnetic resonance imaging in patients recently recovered from COVID-19. JAMA Cardiol. (2020) 5:1265–73. 10.1001/jamacardio.2020.3557 32730619 PMC7385689

[11] Hanneman K Houbois C Kei T Gustafson D Thampinathan B Sooriyakanthan M Multimodality cardiac imaging, cardiac symptoms, and clinical outcomes in patients who recovered from mild COVID-19. Radiology. (2023) 308:e230767. 10.1148/radiol.230767 37432085

[12] Ferreira VM Schulz-Menger J Holmvang G Kramer CM Carbone I Sechtem U Cardiovascular magnetic resonance in nonischemic myocardial inflammation: expert recommendations. J Am Coll Cardiol. (2018) 72:3158–76. 10.1016/j.jacc.2018.09.072 30545455

[13] Cai J Lin K Zhang H Xue Q Zhu K Yuan G A one-year follow-up study of systematic impact of long COVID symptoms among patients post SARS-CoV-2 omicron variants infection in Shanghai, China. Emerg Microbes Infect. (2023) 12:2220578. 10.1080/22221751.2023.2220578 37272336 PMC10281439

[14] Smiseth OA Torp H Opdahl A Haugaa KH Urheim S. Myocardial strain imaging: how useful is it in clinical decision making? Eur Heart J. (2016) 37:1196–207. 10.1093/eurheartj/ehv529 26508168 PMC4830908

[15] Li X Wang H Zhao R Wang T Zhu Y Qian Y Elevated extracellular volume fraction and reduced global longitudinal strains in participants recovered from COVID-19 without clinical cardiac findings. Radiology. (2021) 299:E230–40. 10.1148/radiol.2021203998 33434112 PMC7808090

[16] Puntmann VO Martin S Shchendrygina A Hoffmann J Ka MM Giokoglu E Long-term cardiac pathology in individuals with mild initial COVID-19 illness. Nat Med. (2022) 28:2117–23. 10.1038/s41591-022-02000-0 36064600 PMC9556300

[17] Ibrahim EH Rubenstein J Sosa A Stojanovska J Pan A North P Myocardial strain for the differentiation of myocardial involvement in the post-acute sequelae of COVID-19: a multiparametric cardiac MRI study. Tomography. (2024) 10:331–48. 10.3390/tomography10030026 38535768 PMC10974260

[18] Nedeljkovic-Arsenovic O Ristić A Đorđević N Tomić M Krljanac G Maksimović R. Cardiac magnetic resonance imaging as a risk stratification tool in COVID-19 myocarditis. Diagnostics (Basel). (2024) 14:790. 10.3390/diagnostics14080790 38667436 PMC11049213

[19] Li Z Zhao R Wang C Wang Y Lin J Zhao S Cardiac magnetic resonance-based layer-specific strain in immune checkpoint inhibitor-associated myocarditis. ESC Heart Fail. (2024) 11:1061–75. 10.1002/ehf2.14664 38243390 PMC10966230

[20] Ferreira VM Plein S Wong TC Tao Q Raisi-Estabragh Z Jain SS Cardiovascular magnetic resonance for evaluation of cardiac involvement in COVID-19: recommendations by the society for cardiovascular magnetic resonance. J Cardiovasc Magn Reson. (2023) 25:21. 10.1186/s12968-023-00933-0 36973744 PMC10041524

[21] Popovic M Cvetic V Popadic V Ilic K Radojevic A Klasnja A The correlation between cardiac magnetic resonance findings and post-COVID-19: the impact of myocardial injury on quality of life. Diagnostics (Basel). (2024) 14:1937. 10.3390/diagnostics14171937 39272722 PMC11394307

[22] Wojtowicz D Dorniak K Ławrynowicz M Wąż P Fijałkowska J Kulawiak-Gałąska D Cardiac magnetic resonance findings in patients recovered from COVID-19 pneumonia and presenting with persistent cardiac symptoms: the TRICITY-CMR trial. Biology (Basel). (2022) 11:1848. 10.3390/biology11121848 36552357 PMC9775441

[23] Babapoor-Farrokhran S Gill D Walker J Rasekhi RT Bozorgnia B Amanullah A. Myocardial injury and COVID-19: possible mechanisms. Life Sci. (2020) 253:117723. 10.1016/j.lfs.2020.117723 32360126 PMC7194533

[24] Dhakal BP Sweitzer NK Indik JH Acharya D William P. SARS-CoV-2 infection and cardiovascular disease: cOVID-19 heart. Heart Lung Circ. (2020) 29:973–87. 10.1016/j.hlc.2020.05.101 32601020 PMC7274628

[25] Sozzi FB Gherbesi E Faggiano A Gnan E Maruccio A Schiavone M Viral myocarditis: classification, diagnosis, and clinical implications. Front Cardiovasc Med. (2022) 9:908663. 10.3389/fcvm.2022.908663 35795363 PMC9250986

[26] Barone-Rochette G Augier C Rodière M Quesada JL Foote A Bouvaist H Potentially simple score of late gadolinium enhancement cardiac MR in acute myocarditis outcome. J Magn Reson Imaging. (2014) 40:1347–54. 10.1002/jmri.24504 24293405

[27] Abdeldayem EH Raief Mosaad BM Yassin A Abdelrahman AS. Cardiac MRI in patients with COVID-19 infection. Eur Radiol. (2023) 33:3867–77. 10.1007/s00330-022-09325-x PMC9745285 36512043

[28] Sinclair JE Vedelago C Ryan FJ Carney M Redd MA Lynn MA Post-acute sequelae of SARS-CoV-2 cardiovascular symptoms are associated with trace-level cytokines that affect cardiomyocyte function. Nat Microbiol. (2024) 9:3135–47. 10.1038/s41564-024-01838-z 39478108 PMC11602718

[29] Roca-Fernandez A Wamil M Telford A Carapella V Borlotti A Monteiro D Cardiac abnormalities in long COVID 1-year post-SARS-CoV-2 infection. Open Heart. (2023) 10:e002241. 10.1136/openhrt-2022-002241 36822818 PMC9950586

[30] Breitbart P Koch A Schmidt M Magedanz A Lindhoff-Last E Voigtländer T Clinical and cardiac magnetic resonance findings in post-COVID patients referred for suspected myocarditis. Clin Res Cardiol. (2021) 110:1832–40. 10.1007/s00392-021-01929-5 34448040 PMC8390029

[31] Gyöngyösi M Hasimbegovic E Han E Zlabinger K Spannbauer A Riesenhuber M Improvement of symptoms and cardiac magnetic resonance abnormalities in patients with post-acute sequelae of SARS-CoV-2 cardiovascular syndrome (PASC-CVS) after guideline-oriented therapy. Biomedicines. (2023) 11:3312. 10.3390/biomedicines11123312 38137533 PMC10742066

