# Supplementary material for: Longitudinal assessment of myocardial involvement in PASC-CVS: a single-center study from China based on multiparametric CMR
Source: Front Cardiovasc Med. 2026 May 18;13:1725291. doi: 10.3389/fcvm.2026.1725291 (PMC13223151; doi:10.3389/fcvm.2026.1725291)
Supplement: Supplementary file 1 [file Datasheet1.docx]

Supplementary Material

# Supplementary Figures and Tables

## Supplementary Figures


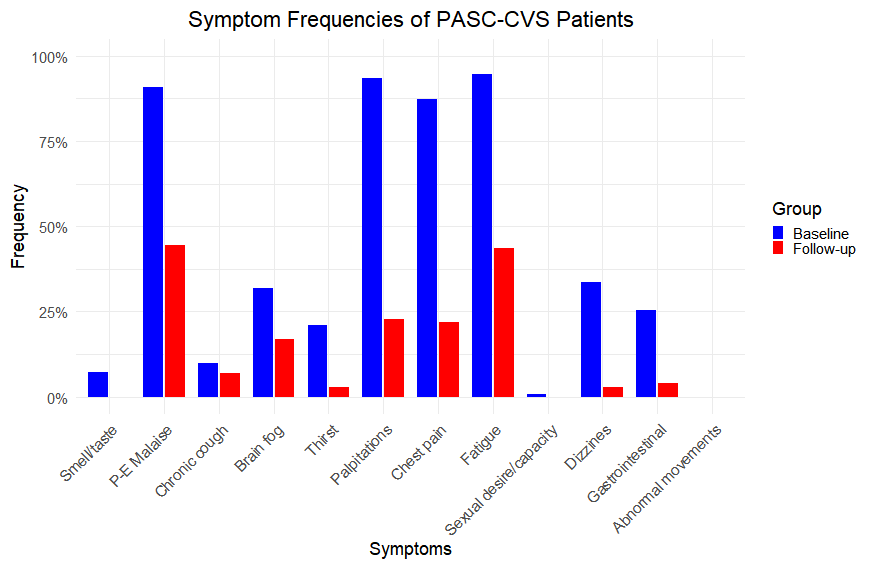


**Supplementary Figure 1.** Symptom frequencies of PASC-CVS patients. The figure displays the 12 clinical symptoms included in the PASC score, encompassing smell/taste, P-E malaise, chronic cough, brain fog, thirst, palpitations, chest pain, fatigue, sexual desire/capacity, dizziness, gastrointestinal issues, and abnormal movements. Baseline data were collected from 110 participants, while follow-up data were obtained from 101 participants after a follow-up period of 10–14 months.


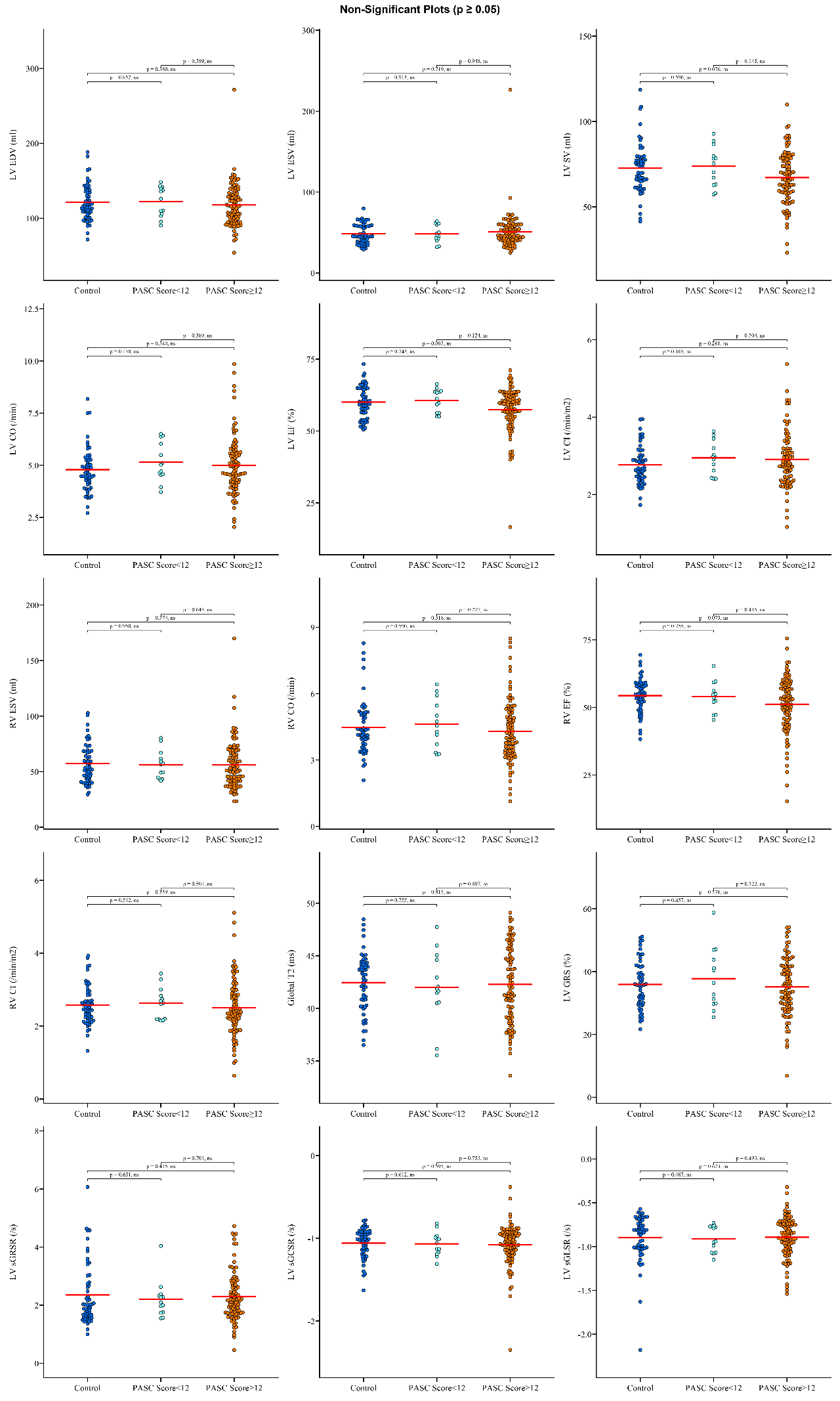


**Supplementary Figure 2.** Comparison of CMR Parameters Among Control, PASC Score < 12, and PASC Score ≥ 12 Groups. Distribution of cardiovascular magnetic resonance (CMR) parameters among three groups: Control (n=55), PASC Score < 12 (n=21), and PASC Score ≥ 12 (n=89).All P values shown are <0.05, indicating statistical significance. Significant differences are marked with asterisks, with the number of asterisks corresponding to the level of significance (e.g., *p < 0.05, **p < 0.01, ***p < 0.001).


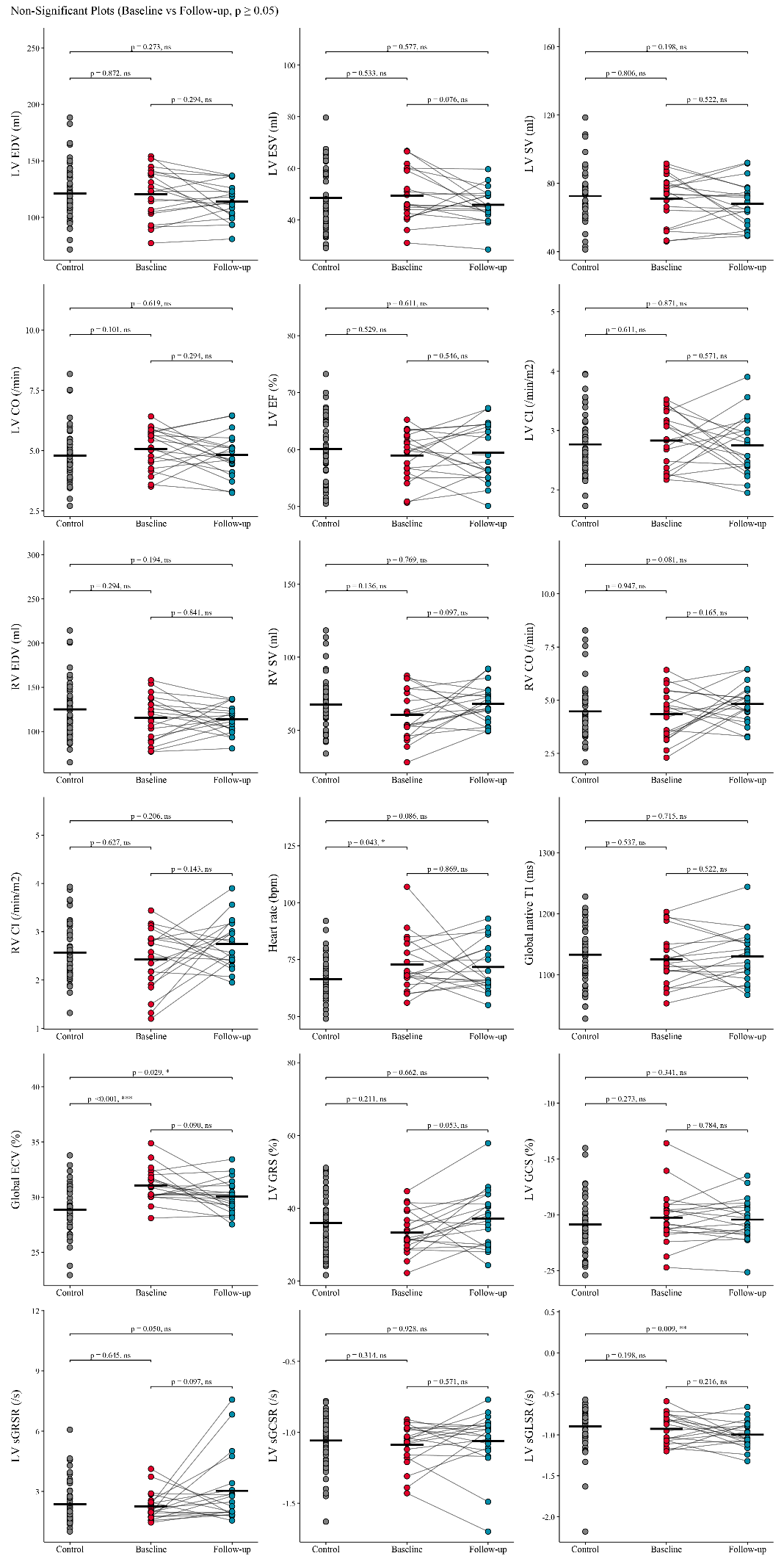


**Supplementary Figure 3.** Comparison of CMR Parameters Among the Control, Baseline, and Follow-up. Distribution of cardiovascular magnetic resonance (CMR) parameters among three groups: Control (n=55), Baseline (n=20), and Follow-up (n=20). All P values shown are <0.05, indicating statistical significance. Significant differences are marked with asterisks, with the number of asterisks corresponding to the level of significance (e.g., *p < 0.05, **p < 0.01, ***p < 0.001). For additional comparisons, see Fig. S2.

## Supplementary Tables

**Supplementary Table 1.** Cardiac MRI Parameters Used in the Study

| Technique | Cine | Late Gadolinium Enhancement | T1-Mapping | T2-Mapping |
| --- | --- | --- | --- | --- |
| Sequence | bSSFP | PSIR | MOLLI | T2-prepared FLASH |
| Parallel Imaging | GRAPPA; R=2 | GRAPPA; R=2 | GRAPPA; R=2 | GRAPPA; R=2 |
| Pixel Dimensions (mm) | 1.70 × 1.70 | 1.98 × 1.48 | 2.12 × 1.48 | 2.20 × 1.98 |
| Slice thickness (mm) | 8 | 8 | 8 | 8 |
| TR/TE (ms) | 2.8/1.28 | 5.16/2.11 | 2.72/1.62 | 2.86/1.31 |
| Miscellaneous | FOV: 380 × 380 mm²  Flip angle: 60° | FOV: 380 × 380 mm²  Flip angle: 18°  Inversion time: 315 ms | FOV: 380 × 380 mm²  Flip angle: 35°  Inversion time: 155 ms | FOV: 380 × 380 mm²  Flip angle: 35° |

For measurement of T1 and T2 relaxation times, source images were examined for artifacts and any segments with artifact were excluded from analysis. All images were obtained with breath-hold techniques. Standard motion correction algorithms were applied to T1/T2 mapping images. FOV, Field of view.

**Supplementary Table 2.** Symptoms considered in analysis

| Symptom short name | Symptom as it appears in the questionnaire |
| --- | --- |
| Smell/taste | Loss of or change in smell or taste |
| P-E Malaise | Post-exertional malaise (Symptoms worse after even minor physical or mental effort) |
| Chronic cough | Persistent (chronic) cough |
| Brain fog | Problems thinking or concentrating ("brain fog") |
| Thirst | Excessive thirst |
| Palpitations | Palpitations, racing heart, arrhythmia, skipped beats |
| Chest pain | Chest pain (including chest tightness, pressure) |
| Fatigue | Fatigue (being very tired) |
| Sexual desire/capacity | Changes in desire for, comfort with or capacity for sex |
| Dizziness | Feeling faint, dizzy, "goofy"; difficulty thinking soon after standing up from a sitting or lying position |
| Gastrointestinal | Gastrointestinal (belly) symptoms (feeling full or vomiting after eating, diarrhea, constipation) |
| Abnormal movements | Abnormal movements |

**Supplementary Table 3.** Baseline Characteristics and One-Year Follow-Up Results of the Study Population

|  | Overall Population Baseline (n=110) | Follow-up Population Baseline (n=101) | Follow-up Population Follow-up (n=101) | *P* value^#^ |
| --- | --- | --- | --- | --- |
| Median Follow-Up Time (days)^#^ | NA | NA | 371.00 (340.00–409.00) | NA |
| PASC Score | 14.09 ± 4.39 | 14.07 ± 4.35 | 5.39 ± 5.11 | <0.001^*^ |
| Improvement（N，%） | NA | NA | 91（90.10） | NA |
| Smell/taste（N，%） | 8（7.27） | 8（7.92） | 0（0.00） | 0.007^*^ |
| P-E Malaise（N，%） | 100（90.91） | 93（92.08） | 45（44.55） | <0.001^*^ |
| Chronic cough（N，%） | 11（10.00） | 9（8.91） | 7（6.93） | 0.795 |
| Brain fog（N，%） | 35（31.82） | 33（32.67） | 17（16.83） | 0.014^*^ |
| Thirst（N，%） | 23（20.91） | 17（16.83） | 3（2.97） | 0.002^*^ |
| Palpitations（N，%） | 103（93.64） | 94（93.07） | 23（22.77） | <0.001^*^ |
| Chest pain（N，%） | 96（87.27） | 88（87.13） | 22（21.78） | <0.001^*^ |
| Fatigue（N，%） | 104（94.55） | 96（95.05） | 44（43.56） | <0.001^*^ |
| Sexual desire/capacity（N，%） | 1（0.91） | 1（0.99） | 0（0.00） | NA |
| Dizziness（N，%） | 37（33.64） | 33（32.67） | 3（2.97） | <0.001^*^ |
| Gastrointestinal（N，%） | 28（25.45） | 26（25.74） | 4（3.96） | <0.001^*^ |
| Abnormal movements（N，%） | 0（0.00） | 0（0.00） | 0（0.00） | NA |

Values are presented as mean ± standard deviation or number (%). #, P values are calculated for the comparison between Follow-up Population Baseline (n=101) and Follow-up Population Follow-up (n=101). This table presents the baseline clinical symptoms and PASC scores of the overall included PASC-CVS patients, as well as the baseline and 10–14 month follow-up clinical symptoms and PASC scores of the 101 patients successfully followed up by telephone.

**Supplementary Table 4.** CMR Findings of Prior-infection Controls and Never-infected Controls

|  | Prior-infection Controls (n=40) | Never-infected Controls (n=15) | P value |
| --- | --- | --- | --- |
| LV EDV（ml） | 122.81 ± 19.94 | 120.56 ± 25.23 | 0.527 |
| LV ESV（ml） | 47.43 ± 11.59 | 48.96 ± 12.44 | 0.755 |
| LV SV（ml） | 75.38 ± 12.23 | 71.60 ± 15.89 | 0.277 |
| LV CO（/min） | 5.01 ± 1.00 | 4.71 ± 1.08 | 0.174 |
| LV EF（%） | 61.66 ± 5.32 | 59.45 ± 5.51 | 0.133 |
| LV CI（/min/m2） | 2.88 ± 0.47 | 2.72 ± 0.49 | 0.146 |
| RV EDV（ml） | 125.32 ± 28.84 | 125.09 ± 31.38 | 0.962 |
| RV ESV（ml） | 57.00 ± 14.18 | 57.59 ± 18.46 | 0.917 |
| RV SV（ml） | 68.32 ± 19.92 | 67.50 ± 16.03 | 0.617 |
| RV CO（/min） | 4.53 ± 1.37 | 4.45 ± 1.18 | 0.685 |
| RV EF（%） | 54.26 ± 7.04 | 54.42 ± 6.09 | 0.828 |
| RV CI（/min/m2） | 2.60 ± 0.65 | 2.56 ± 0.50 | 0.835 |
| Heart rate (bpm) | 66.47 ± 8.63 | 66.39 ± 9.74 | 0.733 |
| Global native T1（ms） | 1118.01 ± 33.44 | 1138.42 ± 53.11 | 0.165 |
| Global post T1（ms） | 401.64 ± 51.24 | 380.36 ± 78.84 | 0.236 |
| Global ECV（%） | 28.38 ± 1.86 | 28.99 ± 2.31 | 0.549 |
| Global T2（ms） | 43.00 ± 2.33 | 42.25 ± 2.88 | 0.33 |
| LV GRS（%） | 38.51 ± 9.29 | 34.94 ± 6.79 | 0.148 |
| LV GCS（%） | -21.16 ± 2.32 | -20.77 ± 2.25 | 0.4 |
| LV GLS（%） | -16.55 ± 2.11 | -16.11 ± 1.81 | 0.539 |
| LV sGRSR (/s) | 2.55 ± 1.10 | 2.28 ± 1.06 | 0.461 |
| LV sGCSR (/s) | -1.09 ± 0.20 | -1.05 ± 0.17 | 0.719 |
| LV sGLSR (/s) | -0.90 ± 0.21 | -0.90 ± 0.30 | 0.551 |
| LGE（N，%） | 3 (30.00) | 14 (38.89) | 0.885 |
| Quantification of LGE (%) | 0.34 ± 0.12 | 0.34 ± 0.17 | 0.862 |

Values are presented as mean ± standard deviation or number (%). No statistically significant differences were observed between the two control subgroups for any CMR parameter (all P > 0.05).

**Supplementary Table 5.** Baseline Characteristics and CMR Findings of Controls, PASC Score < 12, and PASC Score ≥ 12 Groups

|  | Control(n=55) | PASC Score < 12(n=13) | PASC Score ≥ 12(n=97) | *P*^1^ value | *P*^2^ value | *P*^3^ value |
| --- | --- | --- | --- | --- | --- | --- |
| Age（years） | 42.87 ± 13.89 | 39.62 ± 9.00 | 43.06 ± 16.22 | 0.431 | 0.863 | 0.756 |
| Male Gender（N，%） | 25 (45.45) | 4 (30.77) | 35 (36.08) | 0.372 | 0.335 | 1.000 |
| BMI（kg/m2） | 23.52 ± 3.61 | 24.81 ± 3.93 | 22.78 ± 3.51 | 0.300 | 0.232 | 0.056 |
| Smoking history（N，%） | 9 (16.36) | 4 (30.77) | 15 (15.46) | 0.254 | 1.000 | 0.234 |
| Comorbidities |  |  |  |  |  |  |
| Hypertension（N，%） | 8 (14.55) | 1 (7.69) | 16 (16.49) | 1.000 | 0.932 | 0.687 |
| Diabetes（N，%） | 2 (3.64) | 1 (7.69) | 6 (6.19) | 0.477 | 0.711 | 1.000 |
| Hyperlipidemia（N，%） | 4 (7.27) | 1 (7.69) | 13 (13.40) | 1.000 | 0.295 | 1.000 |
| Heart Failure（N，%） | 0 (0.00) | 0 (0.00) | 0 (0.00) | NA | NA | NA |
| Severe coronary artery disease（N，%） | 0 (0.00) | 0 (0.00) | 0 (0.00) | NA | NA | NA |
| Other Heart Diseases（N，%） | 0 (0.00) | 0 (0.00) | 0 (0.00) | NA | NA | NA |
| History of Coronary Artery Surgery（N，%） | 0 (0.00) | 0 (0.00) | 0 (0.00) | NA | NA | NA |
| History of Psychotropic Medication（N，%） | 0 (0.00) | 0 (0.00) | 2 (2.06) | NA | 0.535 | 1.000 |
| Depression（N，%） | 0 (0.00) | 0 (0.00) | 0 (0.00) | NA | NA | NA |
| Anxiety（N，%） | 0 (0.00) | 0 (0.00) | 13 (13.40) | NA | 0.004^*^ | 0.358 |
| Blood biomarkers |  |  |  |  |  |  |
| Troponin-positive（N，%） | NA | 0 (0.00) | 1 (1.03) | NA | NA | 1.000 |
| COVID-19 vaccination |  |  |  |  |  |  |
| At least three dose prior to MRI（N，%） | 54 (98.18) | 13 (100.00) | 86 (88.66) | 1.000 | 0.056 | 0.355 |
| Cardiac MRI |  |  |  |  |  |  |
| Days from virus detection to CMR (days) | NA | 257.46 ± 152.22 | 197.66 ± 105.69 | NA | NA | 0.192 |
| Days from virus detection to CMR (days)^#^ | NA | 173.00 (143.00–387.00) | 168.00 (104.00–256.00) | NA | NA | 0.200 |
| uMR 780（N，%） | 36 (65.45) | 8 (61.54) | 76 (78.35) | 1.000 | 0.123 | 0.321 |
| LV EDV（ml） | 121.17 ± 23.74 | 122.03 ± 20.23 | 117.89 ± 28.02 | 0.904 | 0.380 | 0.389 |
| LV ESV（ml） | 48.54 ± 12.12 | 48.29 ± 10.65 | 50.74 ± 21.73 | 0.913 | 0.719 | 0.948 |
| LV SV（ml） | 72.63 ± 14.97 | 73.75 ± 11.69 | 67.14 ± 15.30 | 0.596 | 0.076 | 0.138 |
| LV CO（l/min） | 4.79 ± 1.05 | 5.16 ± 0.94 | 5.00 ± 1.40 | 0.138 | 0.344 | 0.389 |
| LV EF（%） | 60.05 ± 5.50 | 60.64 ± 3.98 | 57.46 ± 7.65 | 0.717 | 0.093 | 0.124 |
| LV CI（l/min/m2） | 2.77 ± 0.48 | 2.95 ± 0.42 | 2.91 ± 0.71 | 0.218 | 0.281 | 0.504 |
| RV EDV（ml） | 125.15 ± 30.45 | 122.32 ± 22.47 | 113.48 ± 28.54 | 0.938 | 0.022^*^ | 0.287 |
| RV ESV（ml） | 57.42 ± 17.27 | 56.27 ± 12.84 | 56.03 ± 21.76 | 0.950 | 0.374 | 0.643 |
| RV SV（ml） | 67.73 ± 16.99 | 66.05 ± 13.89 | 57.45 ± 16.04 | 0.963 | <0.001^*^ | 0.068 |
| RV CO（l/min） | 4.47 ± 1.22 | 4.63 ± 1.10 | 4.29 ± 1.41 | 0.596 | 0.318 | 0.277 |
| RV EF（%） | 54.38 ± 6.30 | 53.99 ± 5.53 | 51.18 ± 10.28 | 0.840 | 0.073 | 0.415 |
| RV CI（l/min/m2） | 2.57 ± 0.54 | 2.62 ± 0.44 | 2.50 ± 0.78 | 0.750 | 0.359 | 0.501 |
| Heart rate (bpm) | 66.41 ± 9.37 | 70.54 ± 11.83 | 74.92 ± 12.79 | 0.180 | <0.001^*^ | 0.182 |
| Global native T1（ms） | 1132.86 ± 49.10 | 1143.76 ± 67.15 | 1154.84 ± 66.69 | 0.506 | 0.034^*^ | 0.575 |
| Global post T1（ms） | 384.98 ± 73.74 | 454.71 ± 54.69 | 454.75 ± 59.37 | 0.002^*^ | <0.001^*^ | 0.948 |
| Global ECV（%） | 28.86 ± 2.22 | 31.88 ± 2.64 | 31.51 ± 2.59 | <0.001^*^ | <0.001^*^ | 0.629 |
| Global T2（ms） | 42.45 ± 2.74 | 42.00 ± 3.50 | 42.28 ± 3.59 | 0.618 | 0.815 | 0.797 |
| LV GRS（%） | 35.91 ± 7.63 | 37.77 ± 9.66 | 35.11 ± 8.96 | 0.457 | 0.576 | 0.322 |
| LV GCS（%） | -20.87 ± 2.25 | -20.52 ± 2.38 | -19.53 ± 3.17 | 0.357 | 0.006^*^ | 0.553 |
| LV GLS（%） | -16.23 ± 1.89 | -16.61 ± 1.48 | -14.92 ± 2.53 | 0.505 | 0.002^*^ | 0.012^*^ |
| LV sGRSR (/s) | 2.35 ± 1.07 | 2.20 ± 0.64 | 2.30 ± 0.83 | 0.651 | 0.415 | 0.701 |
| LV sGCSR (/s) | -1.06 ± 0.18 | -1.07 ± 0.14 | -1.08 ± 0.24 | 0.612 | 0.595 | 0.753 |
| LV sGLSR (/s) | -0.90 ± 0.27 | -0.91 ± 0.15 | -0.89 ± 0.23 | 0.487 | 0.623 | 0.493 |
| LGE（N，%） | 17.0 (36.96) | 8.0 (61.54) | 71.0 (73.20) | 0.206 | <0.001^*^ | 0.583 |
| Quantification of LGE (%) | 0.34 ± 0.16 | 0.71 ± 0.30 | 0.73 ± 0.26 | <0.001^*^ | <0.001^*^ | 0.948 |
| Distribution of LGE, （N，%） |  |  |  |  |  |  |
| Basal | 5.0 (10.87) | 7.0 (53.85) | 65.0 (67.01) | 0.003^*^ | <0.001^*^ | 0.531 |
| Mid | 13.0 (28.26) | 6.0 (46.15) | 27.0 (27.84) | 0.377 | 1.000 | 0.302 |
| Apical | 1.0 (2.17) | 1.0 (7.69) | 4.0 (4.12) | 0.395 | 1.000 | 0.473 |
| subepicardial | 0.0 (0.00) | 0.0 (0.00) | 0.0 (0.00) | NA | NA | NA |
| epicardial | 0.0 (0.00) | 2.0 (15.38) | 2.0 (2.06) | 0.046^*^ | 1.000 | 0.068 |
| mid-myocardial layer | 0.0 (0.00) | 8.0 (61.54) | 71.0 (73.20) | 0.206 | <0.001^*^ | 0.583 |

Values are presented as mean ± standard deviation or number (%) unless otherwise specified; ^#^, Data are expressed as the median, with the interquartile range in parentheses, for continuous variables; ^*^, P＜0.05 Statistically significant; P1, Statistical difference between Control and PASC Score < 12; P2, Statistical difference between Control and PASC Score ≥ 12; P3, Statistical difference between PASC Score < 12 and PASC Score ≥ 12; Abbreviations are consistent with those used in Table 1.

**Supplementary Table 6.** Results of DeLong Test for Comparison of ROC Curves.

| ROC Curves | P value |
| --- | --- |
| Combined vs Quantification of LGE | 0.002^*^ |
| Combined vs Global ECV | <0.001^*^ |
| Combined vs Heart rate | <0.001^*^ |
| Combined vs LV GLS | <0.001^*^ |
| Combined vs Global post T1 | <0.001^*^ |
| Combined vs Global native T1 | <0.001^*^ |
| Quantification of LGE vs Global ECV | 0.029^*^ |
| Quantification of LGE vs Heart rate | <0.001^*^ |
| Quantification of LGE vs LV GLS | <0.001^*^ |
| Quantification of LGE vs Global post T1 | 0.029^*^ |
| Quantification of LGE vs Global native T1 | <0.001^*^ |
| Global ECV vs Heart rate | 0.278 |
| Global ECV vs LV GLS | 0.050 |
| Global ECV vs Global post T1 | 0.977 |
| Global ECV vs Global native T1 | 0.002^*^ |
| Heart rate vs LV GLS | 0.331 |
| Heart rate vs Global post T1 | 0.249 |
| Heart rate vs Global native T1 | 0.101 |
| LV GLS vs Global post T1 | 0.055 |
| LV GLS vs Global native T1 | 0.555 |
| LV GLS vs Global native T1 | 0.555 |

^*^, P＜0.05 Statistically significant.

**Supplementary Table 7.** Consistency of CMR Parameters intra-observer and inter-observer.

|  | intra-observer | | inter-observer | |
| --- | --- | --- | --- | --- |
|  | ICC | 95%CI | ICC | 95%CI |
| LV EDV（ml） | 0.94 | 0.91-0.96 | 0.95 | 0.93-0.97 |
| LV ESV（ml） | 0.92 | 0.89-0.95 | 0.95 | 0.93-0.97 |
| LV SV（ml） | 0.95 | 0.92-0.96 | 0.90 | 0.85-0.93 |
| LV CO（l/min） | 0.90 | 0.85-0.93 | 0.92 | 0.89-0.95 |
| LV EF（%） | 0.94 | 0.91-0.96 | 0.84 | 0.77-0.89 |
| LV CI（l/min/m2） | 0.92 | 0.90-0.95 | 0.92 | 0.89-0.95 |
| RV EDV（ml） | 0.85 | 0.80-0.90 | 0.88 | 0.83-0.92 |
| RV ESV（ml） | 0.87 | 0.82-0.91 | 0.83 | 0.75-0.88 |
| RV SV（ml） | 0.89 | 0.85-0.92 | 0.79 | 0.69-0.85 |
| RV CO（l/min） | 0.69 | 0.56-0.78 | 0.86 | 0.80-0.91 |
| RV EF（%） | 0.85 | 0.79-0.89 | 0.56 | 0.36-0.70 |
| RV CI（l/min/m2） | 0.97 | 0.96-0.98 | 0.82 | 0.73-0.88 |
| Heart rate (bpm) | 1.00 | 1.00-1.00 | 1.00 | 1.00-1.00 |
| Global native T1（ms） | 0.83 | 0.75-0.88 | 0.97 | 0.96-0.98 |
| Global post T1（ms） | 0.99 | 0.99-0.99 | 0.99 | 0.99-1.00 |
| Global ECV（%） | 0.82 | 0.73-0.88 | 0.83 | 0.75-0.89 |
| Global T2（ms） | 0.94 | 0.91-0.96 | 0.97 | 0.95-0.98 |
| LV GRS（%） | 0.99 | 0.99-1.00 | 0.53 | 0.32-0.68 |
| LV GCS（%） | 0.99 | 0.99-1.00 | 0.97 | 0.96-0.98 |
| LV GLS（%） | 0.99 | 0.99-1.00 | 0.94 | 0.91-0.96 |
| LV sGRSR (/s) | 0.95 | 0.92-0.96 | 0.46 | 0.22-0.63 |
| LV sGCSR (/s) | 0.99 | 0.99-1.00 | 0.95 | 0.92-0.96 |
| LV sGLSR (/s) | 0.99 | 0.99-1.00 | 0.96 | 0.94-0.97 |

Intraclass Correlation Coefficient (ICC) values are presented with 95% Confidence Intervals (CI) to assess the reliability of measurements. Higher ICC values indicate better consistency among repeated measurements. The 95% CI provides a range within which the true ICC value is likely to fall. Abbreviations are consistent with those used in Table 1.
